# Association between Pityriasis Rosea (PR) and HHV-6/HHV-7 Infection: Importance of Sample Selection and Diagnostic Techniques

**DOI:** 10.3390/diagnostics14080843

**Published:** 2024-04-18

**Authors:** Mine Aydin Kurc, Gamze Erfan, Ayse Demet Kaya, Dumrul Gülen, Meltem Oznur, Mehmet Emin Yanik

**Affiliations:** 1 Department of Medical Microbiology, Faculty of Medicine, Tekirdag Namik Kemal University, Tekirdag 59030, Türkiye; dgulen@nku.edu.tr; 2 Department of Dermatology, Faculty of Medicine, Acıbadem University, Istanbul 34752, Türkiye; gamzeerfan@gmail.com; 3 Department of Medical Microbiology, Faculty of Medicine, Istanbul Okan University, Istanbul 34959, Türkiye; ademetkaya@hotmail.com; 4 Department of Pathology, Faculty of Medicine, Tekirdag Namik Kemal University, Tekirdag 59030, Türkiye; meloznur@gmail.com; 5 Clinic of Dermatology, Sancaktepe Region Hospital, Istanbul 34885, Türkiye; meminyanik@hotmail.com

**Keywords:** pityriasis rosea, HHV-6, HHV-7, etiology, PCR, serology

## Abstract

Recent studies have focused on the role of human herpesvirus 6 (HHV-6) and human herpesvirus 7 (HHV-7) in PR etiology with varying results. In our study, with the approach that the discrepancy between the results may be related to the different samples and techniques used, we aimed to clarify the etiology by examining tissue and plasma samples using molecular methods and evaluating the results together with serological parameters. Skin biopsies and plasma samples of twenty-five PR patients were tested to detect HHV-6 and HHV-7 DNA using calibrated quantitative real-time polymerase chain reaction (CQ RT-PCR). IgG and IgM antibodies against HHV-6 and HHV-7 were tested by enzyme-linked immunosorbent assay and indirect immunofluorescence. Of the patient group, 64% were positive for HHV-6 IgG without IgM positivity. HHV-6 DNA was present in seven tissue and ten plasma samples. HHV-7 positivity was 100% and 12% for IgG and IgM antibodies, respectively. HHV-7 DNA was detected in four tissue samples and one plasma sample. Patients with HHV-7 DNA-positive plasma and tissue samples had also HHV-7 IgM antibodies. In conclusion, our results seem to support the role of HHV-6/HHV-7 in the etiology of PR. To clarify the etiology of PR and avoid confusion, the collection of different biological materials simultaneously and the usage of CQ RT-PCR as a diagnostic technique are recommended.

## 1. Introduction

The family of human herpesviruses is subdivided into three families including the α-herpesviruses, the β-herpesviruses, and the γ-herpesviruses based upon similarities in viral genomes and biological behavior. The most recently described human herpesviruses, HHV-6 and HHV-7, are mostly associated with skin disorders and recent studies have focused on their association with pityriasis rosea (PR), an acute, self-limiting exanthematous disease of unknown origin [1,2,3]. 

The role of viruses in the etiology of PR was hypothesized decades ago, in view of the light and electron microscopy observations and increased amounts of activated CD4 T cells and Langerhans cells determined in the dermis. Also, by using different cell lines inoculated with suspensions of scales from PR skin lesions, a specific cytopathic effect has been detected. The viruses implicated in the etiology are picornavirus, togavirus, arenavirus, echovirus, coxsackie virus, influenza and parainfluenza virus, parvovirus B19, cytomegalovirus (CMV), and Epstein–Barr virus (EBV), and, more recently, HHVs have been taken into consideration [4].

HHV-6 and HHV-7 have been the more extensively studied viruses in the etiology of PR. Similarly to other HHVs, they cause a generally clinically inapparent primary infection and establish a latent infection in specific cells. They are commonly acquired in early childhood. Roseola infantum is a common disease of childhood that is seen globally and is caused by infection with HHV-6 or, less frequently, by HHV-7 [4]. Some conflicting results have been reported from different investigators supporting the role of one or both of these viruses as well as many negative reports. The current disagreements may be due to the variability of the diagnostic techniques used in different studies [1,4]. 

Among the diagnostic approaches, CQ RT-PCR, a highly sensitive method to detect viral DNA and compare the viral load of patients and different types of specimens of the same patient, is now available. In addition, antibody assays can show us a previous infection as well as a recent one by determining the IgG and IgM levels [4].

Since checking for viral DNA alone without testing for an antibody response to show infection is insufficient to establish or disprove an association [5], in this study, using the approach that the discrepancy between the study results may be related to the different sample collection methods and techniques used, we planned to test the plasma and tissue samples of the PR patients using CQ RT-PCR for the presence of the HHV-6 and HHV-7 genomes and also the antibodies from blood specimens collected simultaneously to understand the role of these viruses in the etiology. 

## 2. Materials and Methods

### 2.1. Patients

Patients who have applied to the Dermatology Clinics of Tekirdağ Namık Kemal University, Medical Faculty, during a period of two years were included in the study. All the patients were subjected to detailed history taking and an examination. The study group was composed of patients who had herald patch and secondary cutaneous eruptions arising after the herald patch with or without additional symptoms (pruritus etc.) and pre-diagnosed as having PR and objected to histopathological examination for diagnosis. Patients with negative histopathological results for PR receiving oral drugs for treatment or with systemic disease were excluded.

After history taking and physical examination, the symptoms and locations of lesions (herald patch, cutaneous eruptions) were recorded, and the severity of PR was assessed using the pityriasis rosea severity score (PRSS) [6]. The diagnosis of the PR patients was based on clinical features and a histopathological evaluation of lesional skin biopsy samples.

Skin samples, both from a herald patch (H) and eruptions (E) for each PR patient were collected when samples were taken for histopathological evaluation, preserved in PBS, and stored at −20 °C until tested to detect HHV-6 and HHV-7 DNA. 

Blood samples were collected to study the serological parameters of HHV-6, HHV-7, EBV, CMV, and parvovirus B19 and to examine the viral loads of HHV-6 and HHV-7. The collected blood samples were stored at −80 °C, and the biopsy specimens were preserved in PBS and stored at −20 °C until tested. For the detection of genetic material of HHV-6 and HHV-7, polymerase chain reaction (PCR) was performed. 

Ten healthy volunteers were included in the study as the control group. For the healthy volunteers, only the blood samples were collected to examine the presence of HHV-6 and HHV-7 DNA and antibodies against the tested viruses. Signed informed consent forms were taken for inclusion in this study. 

### 2.2. Serological Tests

For the detection of parvovirus B19 (Novagnost Parvovirus B19 IgM and IgG-ELISA; Siemens Healthcare, Erlangen, Germany) and EBV VCA (Novagnost EBV VCA IgM and IgG-ELISA; Siemens Healthcare, Germany) antibodies, enzyme-linked immunosorbent assay (ELISA) tests were used according to the manufacturers’ instructions. The EBV EBNA IgM test was performed using a chemiluminescent microparticle immunoassay (Architect assay, CMIA) and the results were evaluated using the Architect i2000 analyzer (Abbott Diagnostics, Chicago, IL, USA). Serum concentrations of EBV EBNA IgG antibodies were detected by enzyme-linked fluorescent assay (ELFA) using the commercial Vidas^®^ (BioMerieux SA, Marcy-l’Étoile, France) Kits. Also, CMV IgM and IgG antibody assays were performed using the AxSYM instrument (Abbott Laboratories, Abbott Park, IL, USA). All the test results were automatically calculated by the devices.

The serum levels of IgM and IgG antibodies against HHV-6 were detected using enzyme-linked immunosorbent assay (ELISA)/enzyme immunoassay (EIA) commercial kits (ELISA-VIDITEST anti-HHV-6 IgM and anti-HHV-6 IgG, Jesenice, Czech Republic) in accordance with the manufacturer’s recommendations. After adding conjugate and substrate, the results were evaluated using an ELISA reader (Bio-Tek ELX800, Abington, WA, USA).

The serum levels of IgM and IgG antibodies against HHV-7 were detected using indirect immunofluorescence tests with HHV-7 IgM (Code CL 123; Conjugate FITC IgM, for Virus IFA- with Rhodamine and Evans blue, Viramed, Rochester, MN, USA) and IgG (Code IHV701G; IFA Human Herpesvirus 7 (HHV-7) IgG Assay, Viramed, USA) in accordance with the manufacturer’s recommendations.

### 2.3. DNA Extraction and Calibrated Quantitative Real-Time PCR (CQ RT-PCR)

HHV-6 and HHV-7 DNA were detected using PCR with specific primers for HHV-6 and HHV-7 DNA sequences on the plasma and tissue samples of the patients.

DNA isolation of the plasma samples using a Fluorion^®^ i12 Blood DNA Extraction Kit (Iontek, Kâğıthane/İstanbul, Türkiye) and the Fluorion^®^ i12 Extraction System was performed using 200 μL of the samples (Iontek, Türkiye). DNA was isolated from tissue samples using the MagPurix DNA Extraction Kit (Zinexts Life Science Corporation, Taiwan), which was based on magnetic particle separation technology, and was performed using 200 μL of fragmented tissue samples and run on a MagPurix MagPurix^®^ Instrument automated nucleic acid purification system. All the procedures were performed according to the manufacturer’s instructions, and the elution volume was 100 μL. The DNA was stored at −20 °C until further use.

To detect the viral loads for HHV-6 and HHV-7 DNA isolated from the plasma and tissue samples, REALQUALITY RS-HHV 6 (Code RQ-15; AB ANALITICA, Padova, Italy) and REALQUALITY RS-HHV 7 (Code RQ-19; AB ANALITICA, Padova, Italy) kits were used. A total of 1 μL of internal control and 5 μL of DNA were added to 13.5 μL of amplification mix. Distilled water was added to a final volume of 25 μL. The PCR conditions consisted of 1 cycle at 50 °C for 2 min and 95 °C for 10 min, followed by 44 cycles at 95 °C for 15 s and 60 °C for 1 min. The sensitivity of the HHV-6 and HHV-7 kits was 0.82 copies of viral genome/μL and 0.4 copies of viral genome/μL, respectively. The viral loads for the HHV-6 and HHV-7 results were evaluated using a BioRad CFX96 Real-time PCR System (BioRad, Hercules, CA, USA) according to the manufacturer’s recommendations. 

### 2.4. Histopathological Evaluation

Skin biopsies of the patients whose preliminary diagnosis was PR were taken, placed in 10% formalin, and sent to the pathology department. Tissue processing was performed on the biopsy samples obtained after fixation. Tissue sections were prepared from the paraffin blocks, stained with hematoxylin and eosin, and then microscopically examined.

### 2.5. Statistical Analysis

Statistical analysis was performed using the Statistical Packages for the Social Sciences (SPSS) for Windows, version 25.0 (SPSS Inc., Chicago, IL, USA) data analysis program. Numbers and percentages were used to express the descriptive statistics. The Pearson chi-square test was used to compare the proportions among the groups, and the Fisher exact test and independent *t*-test were used when appropriate. The results were evaluated using 95% confidence intervals. A *p* < 0.05 value was accepted as significant.

## 3. Results

In the study, 14 (56%) of the 25 patients with PR were females and 11 (44%) were males, aged between 18 and 59 years (median age: 32.0 years). The control group consisted of five (50%) females and five (50%) males aged between 26 and 66 years (median age: 38.7 years) (*p* = 0.132, *p* = 0.756). 

The PR patients presented the typical clinical signs and symptoms with variable severity. All the cases presented a typical herald patch with slightly elevated scaling borders and central resolution, and the skin eruptions were characterized by oval erythematous-squamous lesions on the trunk (20%) or lower/upper limbs (8%) or both the trunk and limbs (72%). The mean time lapse between the herald patch and eruptions was 11.0 ± 6.89 (2–25) days. Pruritus was present in 80% of the patients with different degrees of severity, as mild—if occurred only intermittently and did not interfere with work or rest—(28%), moderate—if present for much of the day but at a tolerable level—(32%), and severe—if it interfered with daytime activities or sleep—(20%). Other systemic symptoms during the course of their PR were not reported. All the patients defined their complaints as a primary attack and there were no recurrent or persistent cases. The PRSS score of 25 PR patients was <10 in 8 (32%) patients, 10–20 in 11 (44%), 20–30 in 4 (16%), and 30 in 2 (8%) (range 3–30). 

When seasonal variations of PR were examined based on their visit to dermatology clinics, the cases were frequent in the winter (*n*: 10, 40%) and the fall (*n*: 7, 28%) compared to the spring (*n*: 6, 24%) and summer (*n*: 2, 8%) months. The first hospital visit was common in the months of January and September. 

**Figure 1:**
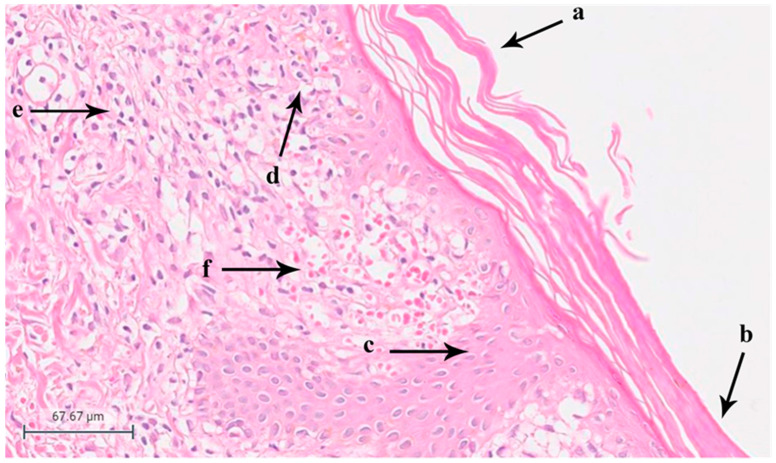
(a) Hyperkeratosis, (b) parakeratosis, (c) irregular acanthosis of the epidermis, (d) acanthosis and spongiosis, (e) perivascular chronic inflammatory cell infiltration in the superficial dermis, (f) erythrocyte extravasation (Skin, 40× magnification, H&E).

A skin biopsy was performed, and the histopathological examination showed hyperkeratosis (*n*: 22), focal parakeratosis (*n*: 16), mild and moderate acanthosis (*n*: 19), spongiosis in the epidermis (*n*: 19), exocytosis (*n*: 18), extravasated red blood cells (*n*: 4), and a perivascular infiltrate of lymphocytes in the upper dermis in all the samples confirming the diagnosis of PR (Figure 1).

The serological parameters for the tested viruses were as follows: for IgG antibodies, CMV was positive in 24 (96%) patients and 9 (90%) controls (*p* = 0.504), EBV-VCA (Viral Capsid Antigen) in 21 (84%) patients and 8 (80%) controls (*p* = 0.784), EBV-EBNA (Epstein–Barr Nuclear Antigen) in 24 (96%) patients and 9 (90%) controls (*p* = 0.504), and parvovirus B19 in 15 (60%) patients and 5 (50%) controls (*p* = 0.602). IgM antibodies against CMV, EBV–VCA, and parvovirus B19 were all negative for the patients and controls (*p* > 0.05) except in one (4%) patient for EBV-EBNA (*p* = 0.535).

In our study, the detected IgG antibodies against HHV-6 were 64% for the patients and 90% for the controls and against HHV-7 was 100% for the patients and controls (*p* = 0.075, *p* > 0.05). IgM antibodies against HHV-6 were not detected in both the patient and control groups, but for HHV-7 IgM, the positivity rate was 12% for the patients and 10% for the controls (*p* > 0.05, *p* = 0.870).

The viral loads for HHV-6 and HHV-7 were analyzed from the tissue (H and E) samples of all the patients and the plasma samples of the patients and controls using CQ RT-PCR. Seven (2H, 5E) tested tissue samples from six (24%) patients showed positive results with a range of 3.39–17.75 viral genome copies/µL, and the plasma samples of 10 (40%) patients with a range of 4.06–22.40 copies/µL yielded HHV-6 DNA positivity. Among the plasma samples, HHV-6 DNA positivity was obtained in ten patient samples, and tissue positivity was present in two patients with high viral loads (tissue: 15.95, plasma: 12.63 and tissue: 17.75, plasma: 22.40 copies/µL) (*p* = 0.999). DNA was positive in all the samples of one patient. In the control group, five (50%) HHV-6 DNA positive (range 3.08–16.31 copies/µL) samples were detected. HHV-7 DNA was negative in the samples of the controls, but in the patient group, DNA positivity was seen in four (16%) of the tissue samples (range 14.44–107.9 copies/µL) and one (4%) of the plasma samples (14.68 copies/µL) (*p* = 0.999). HHV-7 DNA positivity was detected in only one sample of each positive sample. Three samples of the patients with viral loads also had IgM-positive results. Out of 25 PR patients, 4 (16%) showed HHV-6 and HHV-7 DNA viral loads together (Table 1).

## 4. Discussion

Pityriasis rosea typically begins with a single rose-colored, scaling, herald patch followed by secondary eruptions primarily on the trunk, spreading to the limbs. Varying degrees of pruritus can be observed. Classically, there is complete remission mostly within 8–12 weeks [5,7,8]. PR may also have atypical presentations regarding the morphology and distribution of the lesions and the course of the disease. In contrast to the typical PR, relapsing and persistent forms that last longer than 12 weeks have been described in adults and children [9,10]. The recurrence rate was reported as 4.3%; however, the prevalence has probably been underestimated so far [2,11]. Our series contained patients with herald patch and eruptions predominantly localized on both the trunk and limbs, and pruritus was a complaint in 80% of the patients and the severity of the disease was scored by PRRS with a distribution of <10 in 32%, 10–20 in 44%, 20–30 in 16%, and 30 in 8% of the patients. 

The age distribution is generally reported to be between 10 and 35 years of age, similar to the age for primary infection of some viruses, probably due to intimacy during teenage and early adulthood years, which might be related to the spread of the agent [4,7,8]. Although there was a prevalence in women, no statistically significant variation between genders has been claimed [4]. In our study, the patients’ ages were between 18 and 59 years with a median age of 32.0 years, and the difference between the ages and genders was not statistically significant (*p* = 0.132, *p* = 0.756). 

The data available on seasonal variation is also conflicting. Though some epidemiological studies have reported a higher incidence in the colder months and the rainy season, some others reported bimodal distribution or no seasonal variation [5,7,11,12,13]. In our study, the cases were frequent in the winter and fall (68%) compared to the spring and summer.

Many clinical and epidemiological features of PR, like the programmed course with a herald patch and a subsequent eruption followed by spontaneous resolution in weeks with a low risk of recurrence, support an infectious etiology. While seasonal variation, concurrent cases, case clustering, and the observation of virus-like spherical particles on lesional biopsies of the herald patch in electron microscopy studies are the supporting clues, the pathogen has not been identified and real epidemics have not been reported so far. With the present state of knowledge, the benefits of several treatments do not provide strong evidence for or against an infectious etiology [7,14].

The viral etiology for PR was hypothesized decades ago. Arenavirus, echovirus, coxsackie virus, influenza and parainfluenza virus, parvovirus B19, and, more recently, HHVs have been taken into consideration. EBV and CMV, but especially HHV-6 and HHV-7, have been more extensively studied [4]. 

The seroprevalence of CMV varies between countries, increasing with age and higher in countries with low socioeconomic status. In a study conducted in Türkiye, the rate of CMV seropositivity was found to be 97.8% in the 15–49-year age group. Similarly, EBV infections are widespread throughout the world with the rate of seropositivity around 90% in the adult population [15]. For parvovirus B19, the seropositivities of IgG and IgM were reported as 58.8% and 3.9%, respectively [16], and 59.9% for IgG and 0.74% for IgM antibodies [17] in Turkish blood donors. In our study, the seropositivities of the patients and controls were within the previously reported ranges for CMV, EBV, and parvovirus B19, without IgM positivities for each parameter, emphasizing no association of PR with the tested viruses (*p* > 0.05).

HHV-6 and HHV-7 are the viruses commonly acquired in childhood, with an 80% to 90% seroprevalence in the general population, causing clinically unapparent primary infection and latent infection in specific cells, which remains throughout life. HHV-6 and HHV-7 can reactivate in cases of immunosuppression in transplant recipients and during pregnancy and other viral diseases [4,5,7,14,18,19].

For the detection of HHV-6 and HHV-7, viral diagnostic techniques such as DNA/RNA detection, antigen assays, and antibody assays are used. Plasma or serum antibody titers can be detected by indirect immunofluorescent assays or enzyme-linked immunosorbent assays. However, cross-reactivity due to the antigenic similarity between Herpesviridae creates a problem as well as the high seropositivity in the population [5].

In our study, the detected IgG antibodies against HHV-6 were 64% for the patients and 90% for the controls and against HHV-7, they were 100% for both the patients and the controls. IgM antibodies against HHV-6 were not detected in both the patients and the controls, but for HHV-7 IgM, the positivity rate was 12% for the patients and 10% for the controls. IgG seropositivity was similar to the controls (*p* > 0.05) and within the previously reported ranges, but the high prevalence of HHV-6 and HHV-7 infection in the general population poses difficulty in explaining an association if the only parameter that is used is the antibody levels. It is also hard to explain IgM positivity solely without additional tests. Moreover, in serological evaluation, multiple samples are needed to demonstrate seroconversion to prove the active pathological role of the virus. 

To provide sufficient information for a viral etiology, more diagnostic methods need to be performed, such as isolation of the virus, production of a comparable disease in the original host, and reisolation of the virus, which are too time-consuming to have clinical utility and, therefore, researchers have focused on the detection of viral sequences. PBMCs, plasma, and tissue samples are the frequently collected specimens for diagnosis. The usage of different types of multiple biological materials is of importance. 

Besides histopathological evaluation, skin biopsy specimens of PR patients can also be tested by antigen assays, polymerase chain reaction, or, if available, electron microscopy. Qualitative molecular methods, such as nested PCR, are highly sensitive but the detection of viral nucleic acids does not always prove an etiological link as it is not possible to evaluate contamination from latently infected cells and also viral DNA may be below the detection limits in biopsied tissues. When applied to serum samples, nested PCR techniques can be used to detect the viral DNA but provide no information about the viral load [4,5].

CQ RT-PCR is a diagnostic technique that is highly sensitive and facilitates the distinguishing of the viral subtypes and can compare the viral loads of patients and different types of specimens of the same patient. Even 10-genome equivalents/mL of HHV-7 DNA can be quantified with a good level of accuracy and reproducibility without reducing the specificity. This is particularly important in PR, in which HHV-6 and HHV-7 plasma loads are low [4,5,20]. As possible causes of PR, HHV-6 and HHV-7 have been studied most extensively. However, conflicting results have been reported from different investigators. The current variation and disagreement may be due to the variability of the samples and diagnostic techniques. While still controversial, a causative relationship with HHV-6 and HHV-7 seems likely, and it has been suggested that PR is associated with the reactivation of these infections [21,22,23]. 

The results of studies conducted using molecular techniques such as PCR, nested PCR, and RT-PCR are also variable [23]. When skin biopsy samples were studied for HHV-6, Watanabe et al. [21] detected viral DNA in 12 samples out of 14 cases, similar to the positive study results of Drago et al. [24] and Watanabe et al. [21] using nested PCR and Broccolo et al. [22] using real-time PCR. The studies of Selim et al. [14] and Canbolat et al. [3] also showed positivity for HHV-7 DNA in tissue samples with significance. 

In studies conducted to detect the viral genome in PBMCs, positive results were reported by Yasukawa et al. [25], Watanabe et al. [21], and Drago et al. [24] for HHV-6 by PCR and nested PCR.

When plasma was selected as a sample to detect viral DNA, the studies of Drago et al. [24], Watanabe et al. [26], and Watanabe et al. [21] reported positive results by nested PCR, and Broccolo et al. [22] reported positive results by RT-PCR. Mohammed et al. [27] detected HHV-6 and HHV-7 DNA in plasma at a rate of 33 and 54%, respectively. The detection rate for HHV-6 and HHV-7 DNA was reported as 40% and 73.3%, respectively, using a multiplex real-time PCR by Zheng et al. [20]. 

In our study, 10 (40%) plasma samples of the patients and 7 tissue samples of 6 (24%) patients yielded HHV-6 DNA positivity. Higher viral loads were detected in patients with both plasma and tissue positivity. As all the patients were IgG positive, the detected DNA positivities are interpreted as reactivation. HHV-7 DNA was detected in four tissue samples and one plasma sample in the current study. One plasma and two tissue samples in which HHV-7 DNA was detected also had IgM seropositivity, which is also a sign of active infection (*p* < 0.001). These results highlight the importance of testing different types of samples simultaneously in the evaluation of results to understand the link between the agents and the disease.

The limitations of our study include a lack of follow-up studies for the positive patients by means of evaluating the serological results and tissue and blood samples using PCR in addition to not obtaining tissue samples from healthy subjects. If these parameters were also present, the interpretation of the results would be easier. 

## 5. Conclusions

In conclusion, our results seem to support the role of HHV-6 and HHV-7 in the etiology of PR. We used different types of biological materials and also detected an antibody response that could facilitate interpretations. For HHV-6, the resulting IgG positivity in all cases and the detection of viral DNA in plasma with predominance, as well as in tissue samples, were accepted as the signs of reactivation. For HHV-7, while IgG positivity was 100%, the positive IgM result accompanying tissue and plasma sample DNA positivity was also accepted as a sign of reactivation and active infection. Considering these results, we propose the collection of different biological materials simultaneously, following up the patients with positive results, retesting the samples with PR and serology, and interpreting the results with the antibody response. As a diagnostic technique, CQ RT-PCR, which is highly sensitive and can compare the viral loads of patients, is recommended to avoid confusion due to differences in results, which, in turn, may clarify the etiology of PR.

## Figures and Tables

**Table 1:** Distribution of antibodies and viral loads of the patients’ samples.

Patient	Age/ Gender	HHV-6					HHV-7				
		Serology		DNA Copies/μL			Serology		DNA Copies/μL		
		IgG	IgM	E	H	P	IgG	IgM	E	H	P
1	23/F	+	−	N/A	N/A	6.70	+	−	N/A	N/A	N/A
2	49/E	−	−	N/A	N/A	N/A	+	−	N/A	N/A	N/A
3	25/F	+	−	N/A	7.13	N/A	+	−	N/A	N/A	N/A
4	42/F	+	−	7.12	N/A	N/A	+	+	23.44	N/A	N/A
5	39/F	+	−	N/A	N/A	5.89	+	−	N/A	14.62	N/A
6	40/M	+	−	5.87	15.95	12.63	+	−	N/A	N/A	N/A
7	31/F	+	−	5.42	N/A	N/A	+	−	N/A	N/A	N/A
8	25/M	+	−	N/A	N/A	5.98	+	−	N/A	N/A	N/A
9	19/F	+	−	N/A	N/A	6.27	+	−	14.44	N/A	N/A
10	31/M	+	−	N/A	N/A	10.60	+	−	N/A	N/A	N/A
11	42/M	−	−	N/A	N/A	N/A	+	−	N/A	N/A	N/A
12	18/M	+	−	N/A	N/A	5.80	+	−	N/A	N/A	N/A
13	49/M	−	−	N/A	N/A	N/A	+	−	N/A	N/A	N/A
14	29/M	−	−	N/A	N/A	N/A	+	−	N/A	N/A	N/A
15	21/F	−	−	N/A	N/A	N/A	+	−	N/A	N/A	N/A
16	24/F	+	−	N/A	N/A	4.06	+	−	N/A	N/A	N/A
17	22/F	−	−	N/A	N/A	N/A	+	−	N/A	N/A	N/A
18	59/M	+	−	N/A	N/A	N/A	+	−	N/A	N/A	N/A
19	34/M	−	−	3.39	N/A	N/A	+	−	N/A	N/A	N/A
20	18/F	+	−	N/A	N/A	12.25	+	+	N/A	107.9	N/A
21	29/F	+	−	N/A	N/A	N/A	+	−	N/A	N/A	N/A
22	33/F	+	−	N/A	N/A	N/A	+	−	N/A	N/A	N/A
23	45/F	−	−	N/A	N/A	N/A	+	+	N/A	N/A	14.68
24	33/M	−	−	N/A	N/A	N/A	+	−	N/A	N/A	N/A
25	20/F	+	−	17.75	N/A	22.40	+	−	N/A	N/A	N/A

E: Eruption, H: Herald Patch, P: Plasma.

## Data Availability

All the data used to support the findings of this study are included within the article.
